# Spatial and temporal distribution of *Scirtothrips dorsalis* (Thysanoptera: Thripidae) and their natural enemies in Florida strawberry fields

**DOI:** 10.1093/jee/toae111

**Published:** 2024-05-27

**Authors:** Gagandeep Kaur, Lukasz L Stelinski, Xavier Martini, Nathan Boyd, Rachel Mallinger, Sriyanka Lahiri

**Affiliations:** Corteva Agriscience, Indianapolis, IN, USA; Entomology and Nematology Department, Citrus Research and Education Center, University of Florida, Lake Alfred, FL, USA; Entomology and Nematology Department, North Florida Research and Education Center, University of Florida, Quincy, FL, USA; Horticultural Sciences Department, Gulf Coast Research and Education Center, University of Florida, Wimauma, FL, USA; Entomology and Nematology Department, University of Florida, Gainesville, FL, USA; Entomology and Nematology Department, Gulf Coast Research and Education Center, University of Florida, Wimauma, FL, USA

**Keywords:** chilli thrips, spatial distribution, geospatial analysis, border effect

## Abstract

Given the recent invasion of *Scirtothrips dorsalis* Hood in North America, there is limited information regarding their distribution and population dynamics in cultivated small fruit crops. Therefore, we investigated the spatial and temporal distribution of *S. dorsalis* and their natural enemies in commercially produced strawberry fields in Florida. During 2 consecutive strawberry production seasons, 4 and 6 geographically separated strawberry fields were sampled and were divided into grids with 30–40 sampling points per field. At each sampling point, 4–5 leaf and flower samples were collected, and sticky traps were deployed. We quantified the occurrence of *S. dorsalis* as well as potential natural enemies, including *Orius* spp., *Geocoris* spp., and other predators such as long-legged flies. During both years, most of the *S. dorsalis* and natural enemies were found on field borders, and counts progressively diminished further into the interiors of plots and away from field edges. Cluster and outlier analysis revealed that *S. dorsalis* formed statistically significant clusters and that these “hot spots” remained in the same general locations throughout the season. There was a strong relationship between the occurrence of natural enemies and the presence of *S. dorsalis*, but the number of natural enemies was generally low compared to *S. dorsalis*. Our results indicate that targeting field borders for chemical control or planting strawberries away from natural areas containing potential alternative hosts for thrips may be an effective strategy for reducing agricultural inputs; however, future field assessments are needed to determine if these methods could replace the treatment of entire fields.

## Introduction

*Scirtothrips dorsalis* Hood (Thysanoptera: Thripidae) is an invasive pest in the southeastern United States, introduced from Southeast Asia through imported transplants, cut flowers, and fruits (Kumar et al. 2013). The first report of *S. dorsalis* establishment in Florida was in 2005 on rose plants, *Rosa* X “Radrazz” (Rosaceae) (Kumar et al. 2013). Since then, *S. dorsalis* has become an economically important pest of several fruits, flowers, vegetables, and landscape plants while aggressively expanding its invasive range within the United States and on the island of Tenerife, Canary Islands, Spain (Kumar et al. 2013, 2023, Mouratidis et al. 2023). In 2015, *S. dorsalis* was first reported feeding on strawberry plants *Fragaria* × *ananassa* Duchesne (Rosales: Rosaceae) (Panthi et al. 2021). Since then, *S. dorsalis* has emerged as a major pest of strawberry production due to its aggressive feeding, rapid reproduction and development, and ability to escape detection because of its small size and occurrence in cryptic locations during early stages of plant development with injury often going unnoticed (Mound and Teulon 1995, Morse and Hoddle 2006, Kumar et al. 2016).

In Florida, strawberries are grown as an annual crop that is transplanted on fumigated, raised beds, fitted with central drip tape, and covered with black plastic mulch that creates totally impermeable films. These beds are typically ~20 cm tall and 80 cm wide at the base (Dash et al. 2023). Two rows of plug or bare-root strawberry transplants are delivered from nurseries in California and Canada and planted in this manner during mid-late September (early planting) or mid-October (traditional planting date) each year (Dash et al. 2020, 2023, Guan et al. 2020). The bare-root transplants receive overhead irrigation for 10–12 days to alleviate heat stress, after which the drip tape is used to continue fertigation throughout the fruiting season between November and March (Dash et al. 2022). Monitoring for arthropod pests and diseases begins immediately after the overhead irrigation period has been completed, and insecticides are applied as needed, depending on the pest pressure through March (Lahiri 2023). Florida is the only producer of winter strawberries in the United States, accounting for a significant annual production of 4,208 ha with an annual value exceeding $511 million (USDA NASS 2023). Severe infestations by *S. dorsalis* can cause significant yield losses (Lahiri and Panthi 2020).

Insecticides can be effective management tools against *S. dorsalis* in Florida strawberries. For instance, spinetoram, cyantraniliprole, and acetamiprid effectively suppress *S. dorsalis* populations in pepper and strawberry (Seal et al. 2006, Lahiri and Panthi 2020). However, use restrictions allow only 1 or 2 applications of available insecticides against *S. dorsalis* during a 5-month-long strawberry growing season where *S. dorsalis* are present throughout the season (Whitaker et al. 2020, Kaur and Lahiri 2022). *Scirtothrips dorsalis* infestations begin at the start of the strawberry growing season, and if populations are not suppressed initially, they can quickly increase to population levels that are difficult to suppress later in the season (Seal and Klassen 2005, Lahiri and Yambisa 2021). Furthermore, given the lack of other management options, growers rely almost exclusively on insecticides for thrips management, which can result in the emergence of resistant populations (Kaur et al. 2023). In other thrips species, resistance has emerged as a significant problem; for example, *Frankliniella occidentalis* Pergande (Thysanoptera: Thripidae) is resistant to organophosphates, pyrethroids, and carbamates (Zhao et al. 1995, Jensen 2000). Susceptibility changes among *S. dorsalis* populations to commercially used insecticides may change rapidly, given the rapid generation time in this species (Kaur et al. 2023). Therefore, there is a need for integrating additional management tools for *S. dorsalis* such as augmentation of predatory mite populations.

In addition to the integration of multiple management tools, another important factor in *S. dorsalis* management is early diagnosis of infestation so that management tools can be deployed before injury results in economic damage. Until now, the seasonal distribution patterns of *S. dorsalis* within strawberry fields in Florida were unknown. Understanding the timing and distribution of this pest should help time management sprays and may allow for tailored management applications to specific areas of infestation. Therefore, the main objectives of our study were to (i) quantitatively describe the spatial and temporal distribution of *S. dorsalis*, including hot spots, in strawberry fields throughout the season using geospatial tools and (ii) determine if there is a relationship between occurrence of *S. dorsalis* and natural enemies present in strawberry fields.

## Materials and Methods

### *Scirtothrips dorsalis* Distribution and Movement Patterns into Strawberry Fields

#### Study sites.

*Scirtothrips dorsalis* monitoring occurred in 4 conventional strawberry fields (approximately 10–13 ha per field) between 2019 and 2020 and 6 conventional strawberry fields during 2020–2021 (approximately 10–13 ha per field) in Hillsborough County, FL. One of the sites monitored in 2019–2020 was excluded from the analysis because no thrips were captured at that location. Five of the locations were adjacent to natural areas of uncultivated land with significant plant growth and 1 field was adjacent to urbanized land without significant alternative or nonhost plants (Table 1). All strawberry fields were managed under standard agricultural practices for this crop, including irrigation, fertilization, and application of insecticides (Whitaker et al. 2023). Each field was maintained by the growers and received approximately 5–10 management sprays to suppress *S. dorsalis* during November, December, and January. Foliar sprays for *S. dorsalis* management occurred by rotating (in no particular order) spinosyn, diamide, neonicotinoid, and pyrethroid chemical classes added with the insect growth regulator novaluron (Lahiri 2023).

**Table 1.** Description of strawberry fields sampled for *S. dorsalis* and natural enemies during 2019–2020 and 2020–2021 including field coordinates, strawberry cultivars planted, and the surrounding landscape present

Field names	Year sampled	Field coordinates (latitude, longitude)	Cultivars	Surrounding area
Field B	2019–2020, 2020–2021	28.0404, −82.1091	Brilliance, sensation	Barren land
Field C	2019–2020, 2020–2021	28.0619, −82.1218	Brilliance	Woodland and weeds
Field D	2019–2020, 2020–2021	27.9251, −82.0796	Brilliance, sensation	Blueberry field, woodland, and weeds
Field F	2019–2020, 2020–2021	27.9523, −82.1234	Brilliance, sensation	Road, woodland, and weeds
Field G	2020–2021	27.981556, −82.18235	Brilliance, sensation	Woodland and weeds, blueberry field

#### Field layout and sampling.

**Fig. 1.**
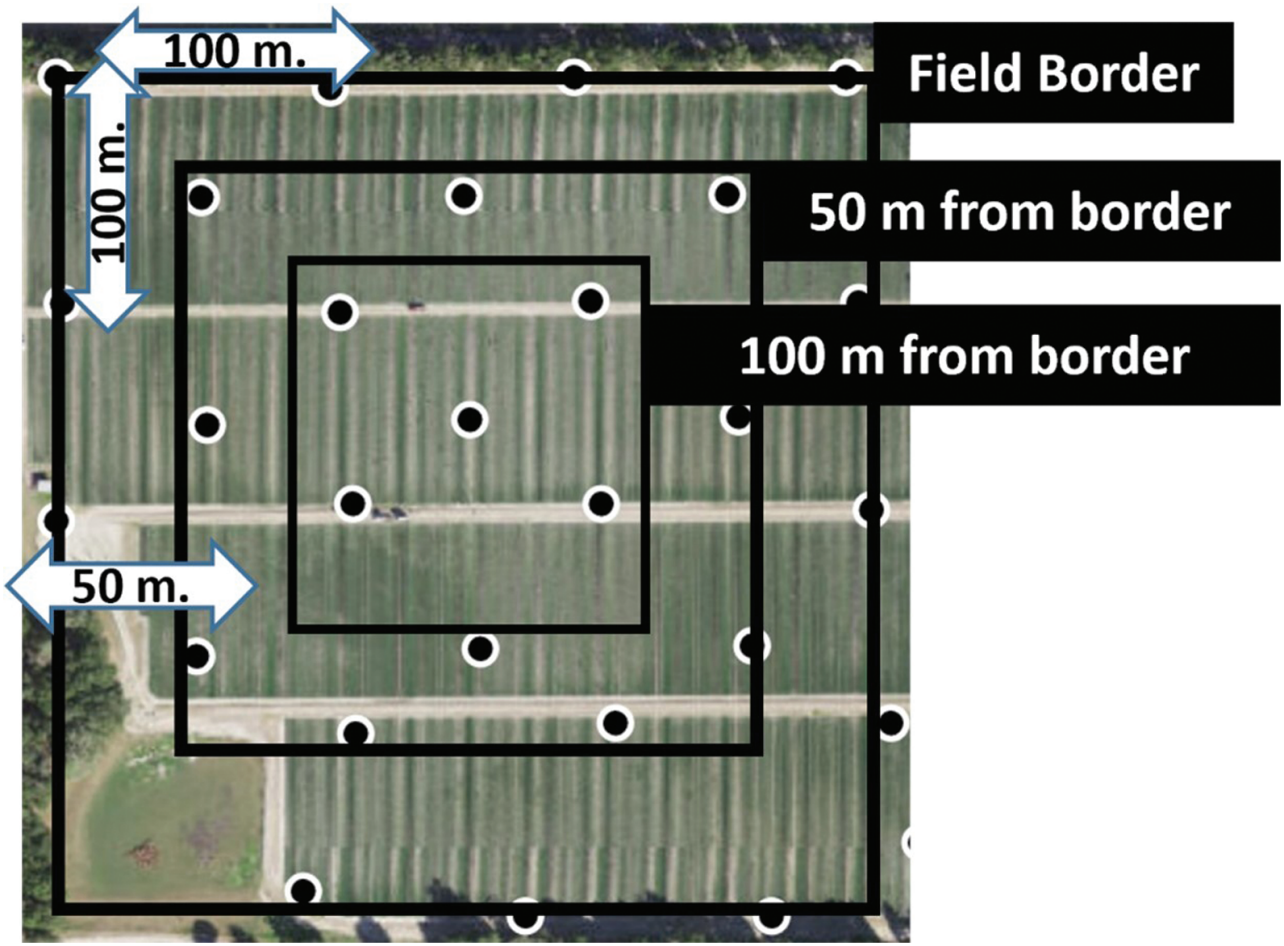
Schematic example of the sampling design for 1 replicate strawberry field in 2019–2020 and 2020–2021. The sampling design was consistent between fields.

To monitor thrips distribution, 30–40 sampling points were established within each field. The sampling points were divided into 3 categories based on location within the field: (i) field border row, (ii) 50-m, and (iii) 100-m inward from the field border. The field border sampling points were 100 m apart from one another, while interior sampling points were laid out in a concentric orientation, either 50- or 100-m inward from borders (Fig. 1). Within each concentric square relative to the plot borders, sampling sites were separated by 100 m, and there was 50 m separation between sampling points in different concentric squares (Fig. 1). The geographic coordinates were noted for identification purposes and data analysis. The sampling was conducted biweekly from October to February. In 2019–2020, 5 leaf trifoliates were collected from nearby rows at each sampling point and date. In 2020–2021, 5 leaf trifoliates and 5 flowers were collected from nearby rows at each sampling point and date. All samples were transported to the laboratory where leaf and flower samples were washed with 70% ethanol (Thermo Fisher Scientific, Hampton, NH, USA) solution and thrips were counted and identified to species level using taxonomic keys (Cluever and Smith 2017).

#### *Natural* e*nemy* o*ccurrence and* d*istribution in* s*trawberry* f*ields.*

Potential natural enemies of thrips were monitored concurrently with thrips. All natural enemies found in foliar and flower samples were counted following the collection procedures described above. Also, trapping was conducted as an additional method of assessing natural enemy populations. Yellow sticky cards (9.8 × 7.3 cm; Faicuk Inc., Las Vegas, NV, USA) were installed and replaced every week at each sampling point in each field (Kumar et al. 2013). Sticky cards were replaced weekly, and collected traps were sealed in plastic bags for transport to the laboratory. All natural enemies of thrips found on traps were counted and identified with the aid of a stereomicroscope to the genus level.

## Data Analysis

### Seasonal Distribution of *Scirtothrips dorsalis* and Natural Enemies in Strawberry

The number of *Scirtothrips dorsalis* and selected natural enemy taxa were analyzed separately for each year by 2-way analysis of variance (ANOVA) across fields using the MIXED procedure of Statistical Analysis Software version 9.4 (SAS Institute, Cary, NC, USA). Trap location within fields (border, 50-m inward from border, and 100-m inward from border) was considered as one of the fixed effects along with the sampling date within each season and the interaction between location and date. Each of the sampled strawberry fields was considered as a block, and block was included as a random effect in model. Mean separation was conducted using Tukey–Kramer HSD tests when significant main and interaction effects between sampling locations and sampling dates were discernible at the 0.05 probability level. Pearson linear correlation within fields was performed to determine the relationships between *S. dorsalis* abundance and the abundance of natural enemies.

### Spatial Distribution of *S. dorsalis* and Identification of “Hot Spots”

The degree of spatial dependence of *S. dorsalis* for each sampling date was analyzed using inverse distance weighting (IDW) and Local Moran’s I index method (Anselin 1995, ArcMap version 10.4.1 Environmental Systems Research Institute 2015). This allowed for the identification of statistically significant clustering tendencies of thrips in the strawberry fields throughout the crop season. This analysis categorizes statistically significant sampling locations into 1 of 4 bins: (i) high–high cluster, (ii) high–low outlier, (iii) low–high outlier, and (iv) low–low cluster. For instance, a significant high–high cluster at a sampling location is interpreted to indicate that the mean number of *S. dorsalis* was high at the sampled location and is also surrounded by sampling locations characterized by high *S. dorsalis* counts. Conversely, a high–low outlier is interpreted to indicate that the mean number of *S. dorsalis* was also high at the sampled location but surrounded by sampling locations characterized by low *S. dorsalis* counts.

## Results

### *Scirtothrips dorsalis* Occurrence Across Time and Space

**Fig. 2.**
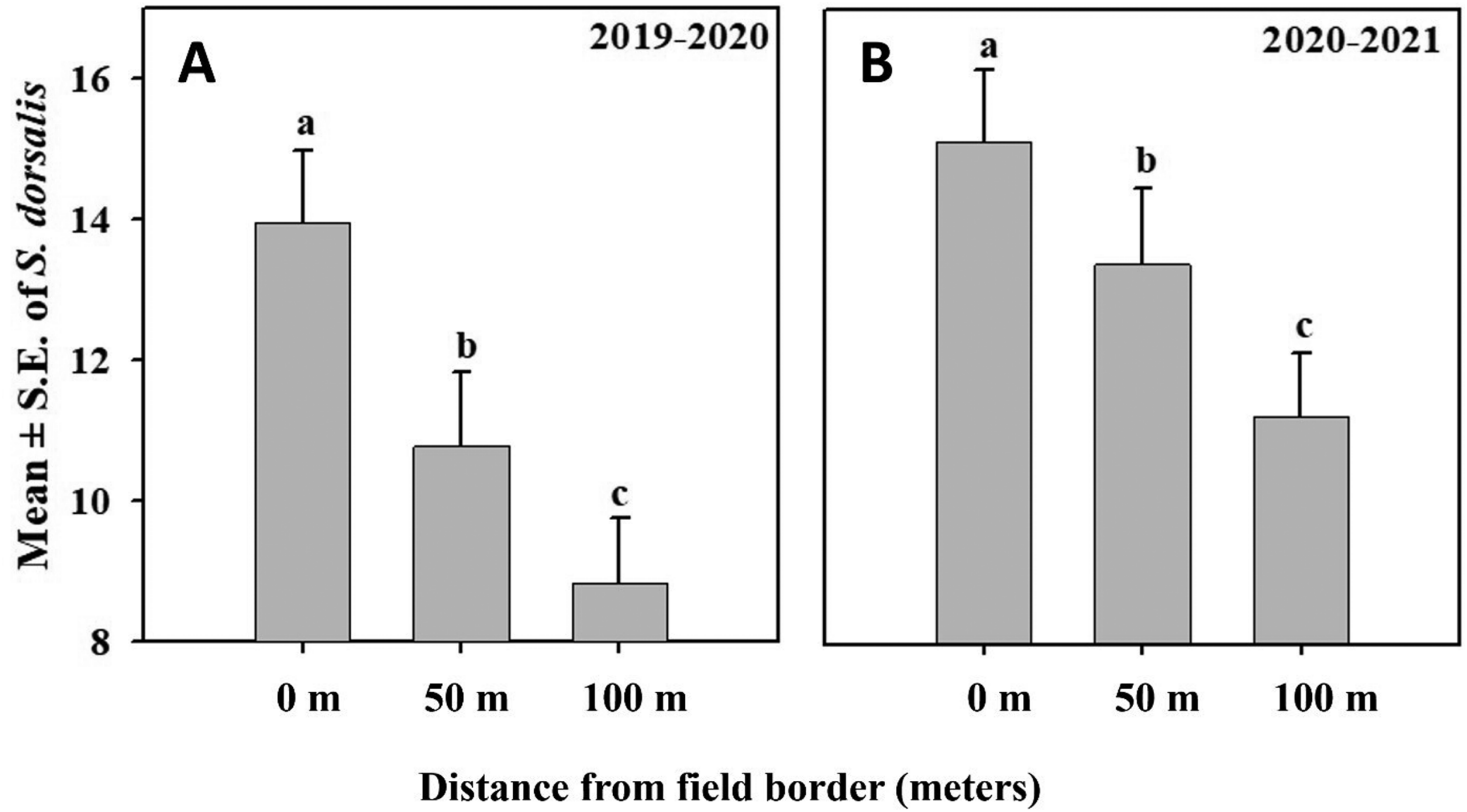
Mean number of *S. dorsalis* captured at 3 different distances relative to field borders (0, 50, and 100 m) during A) 2019–2020 and B) 2020–2021. Columns with different letters differ significantly at *P* < 0.05.

There was a significant effect of sampling location within the field (0, 50, and 100 m from field border) and sampling date on mean number of *S. dorsalis* counted during both the 2019–2020 (*F*_2,78_ = 29.53, *P* = <0.0001; *F*_2,78_ = 32.87, *P* = <0.0001, respectively) and 2020–2021 sampling seasons (*F*_2,104_ = 24.07, *P* = <0.0001; *F*_8,104_ = 77.39, *P* = <0.0001, respectively). However, the interaction between sampling location and sampling date was nonsignificant for 2019–2020 and 2020–2021 (*F*_16,78_ = 0.34, *P* ≤ 0.9915; *F*_16,104_ = 0.36, *P* ≤ 0.9880, respectively). During both years, the mean number of *S. dorsalis* on the field border was significantly higher than at locations 50- and 100-m inward from the border, and significantly more *S. dorsalis* were found 50- than 100-m inward from plot borders (Fig. 2A and B).

**Fig. 3.**
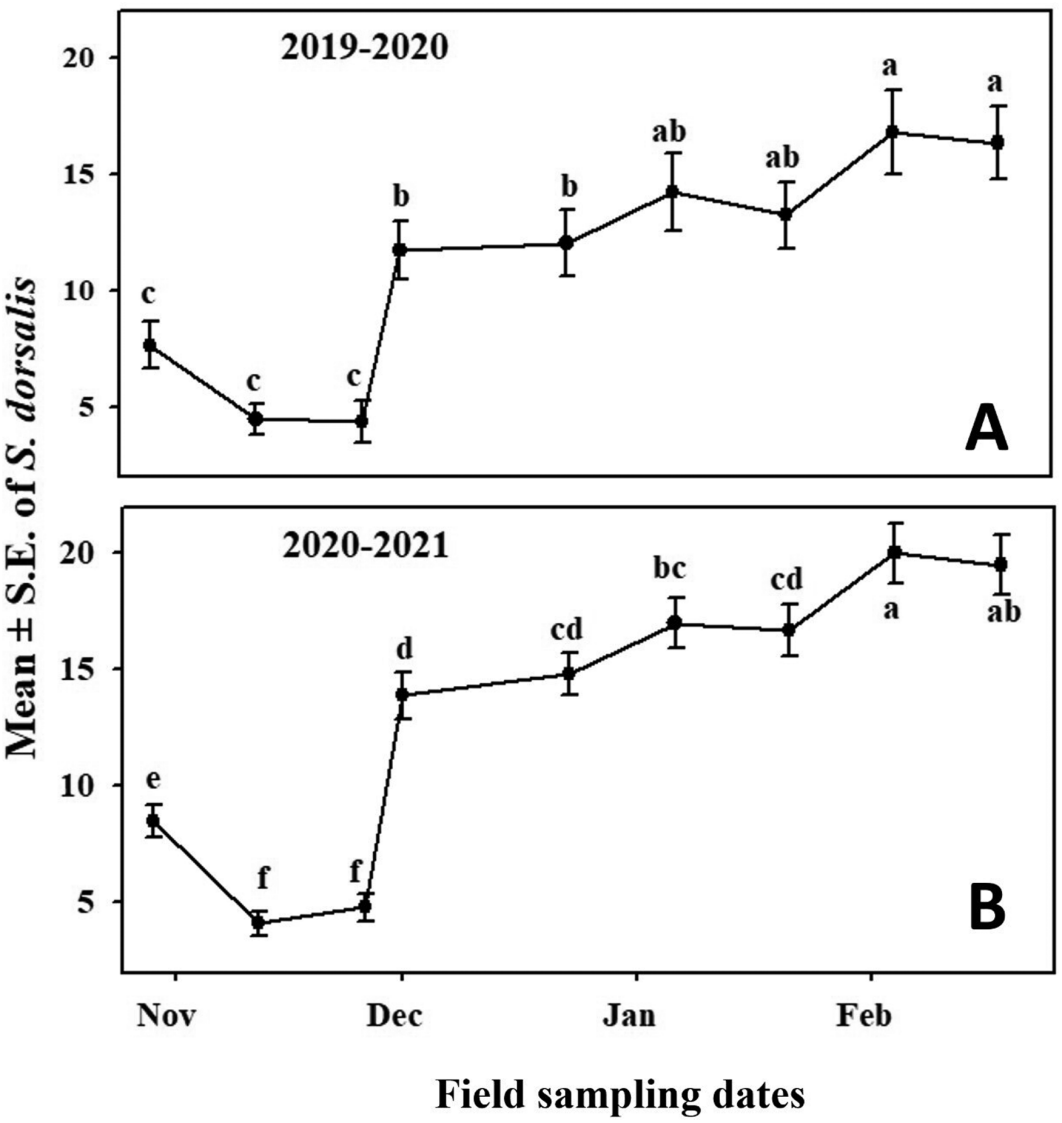
Overall mean number of *S. dorsalis* sampled at 5 commercial strawberry fields in Florida during 2019–2020 and 2020–2021. Dates with different letters differ significantly at *P* < 0.05.

During 2019–2020, significantly more thrips were counted per week during January and February 2020 than during the previous months in October–December 2019 (Fig. 3). During 2020–2021, the seasonal population trend was remarkably similar to that of the previous year. The number of adult *S. dorsalis* counted per week initially decreased significantly from November to December when populations were at their lowest. However, mean weekly counts sharply increased in January and reached a peak by February of 2021 (Fig. 3). During both years, the population reached its lowest point in November/early December, and counts were approximately twice as high during the population peaks in February.

### Natural Enemy Occurrence Across Time and Space

Natural enemies found on yellow sticky cards, strawberry trifoliate, and flower samples belonged to 3 main groups: minute pirate bug, *Orius* spp. Wolff (Hemiptera: Anthocoridae), big-eyed bugs, *Geocoris* spp. (Hemiptera: Geocoridae), and long-legged flies (Diptera: Dolichopodidae). Since low numbers of natural enemies were captured overall, the natural enemy data were pooled into these 3 taxonomic categories, which accounted for the majority of predatory arthropods found on traps.

Like *S. dorsalis*, there was a significant effect of sampling location and sampling date on natural enemy counts in 2019–2020 (*F*_2,78_ = 26.17, *P* ≤ 0.0001; *F*_2,78_ = 57.13, *P* ≤ 0.0001, respectively), and 2020–2021 (*F*_2,104_ = 20.13, *P* ≤ 0.0001; *F*_8,104_ = 78.54, *P* ≤ 0.0001, respectively). However, the interaction between sampling location and sampling date was nonsignificant in both 2019–2020 and 2020–2021 (*F*_16,78_ = 1.27, *P* = 0.2385; *F*_16,104_ = 0.79, *P* ≤ 0.6989, respectively).

**Fig. 4.**
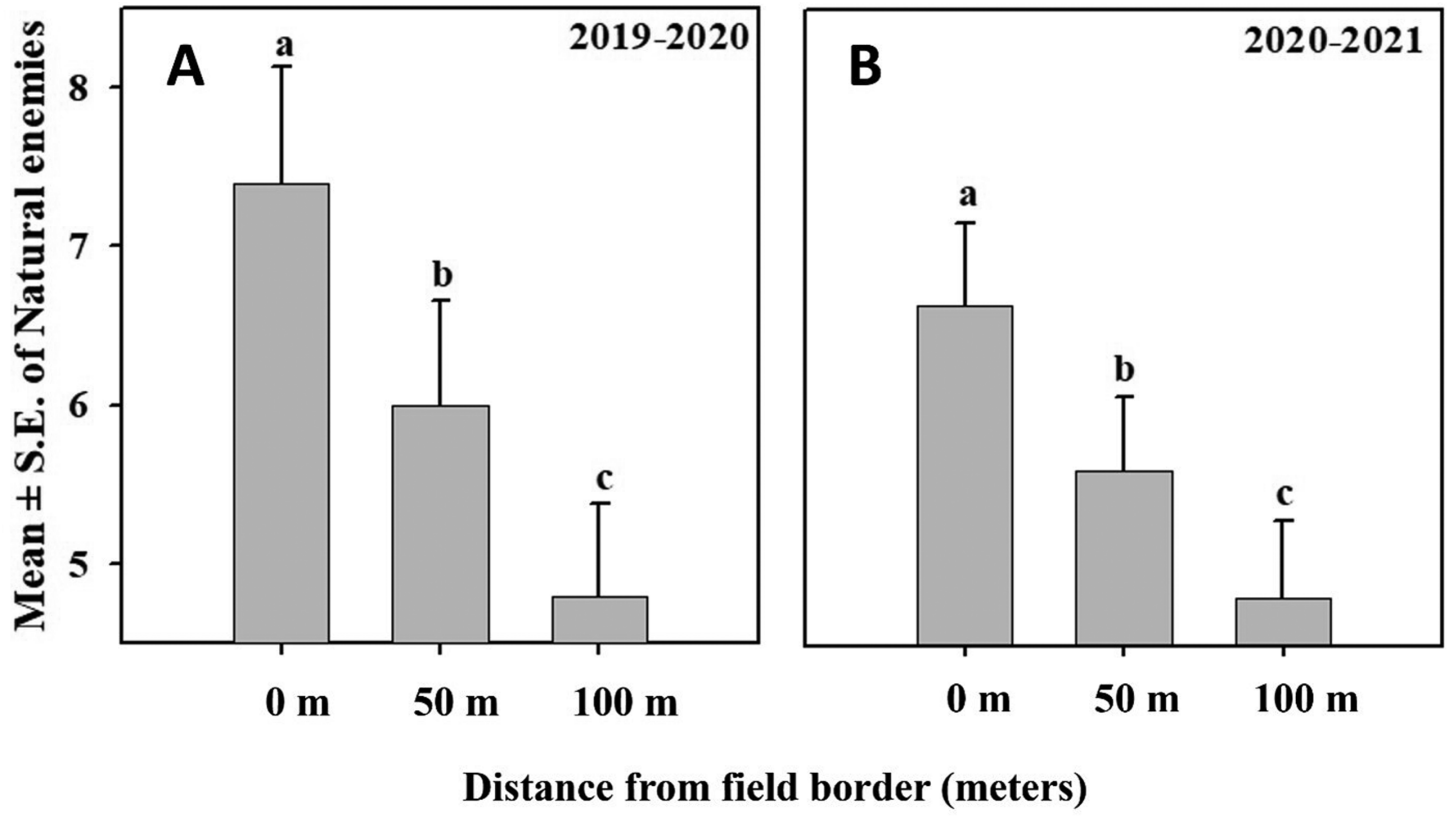
Mean number of natural enemies captured at 3 different distances relative to field borders (0, 50, and100 m) during A) 2019–2020 and B) 2020–2021. Columns with different letters differ significantly at *P* < 0.05.

During both years, significantly more natural enemies were captured on field borders than at locations 50- and 100-m inward from the border, and significantly more predators were found 50- than 100-m inward from plot borders (Fig. 4A and B).

### Correlation Between Natural Enemies and *S. dorsalis* Populations

**Fig. 5.**
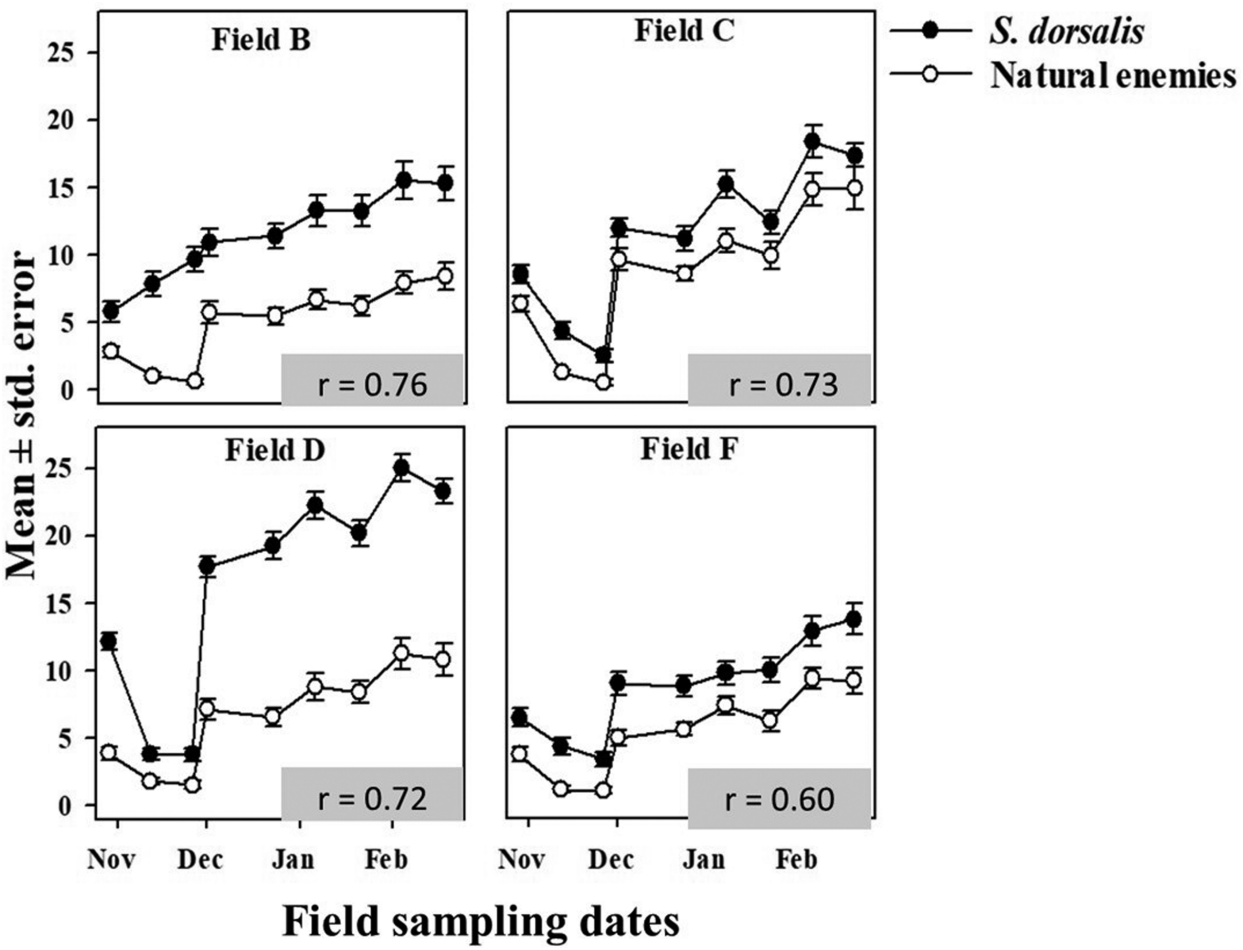
Mean number of *S. dorsalis* and natural enemies counted at 4 sampled commercial strawberry fields in Florida during 2019–2020. Inset *r* values describe correlation between average weekly captures of natural enemies and mean weekly counts of *S. dorsalis*.

**Fig. 6.**
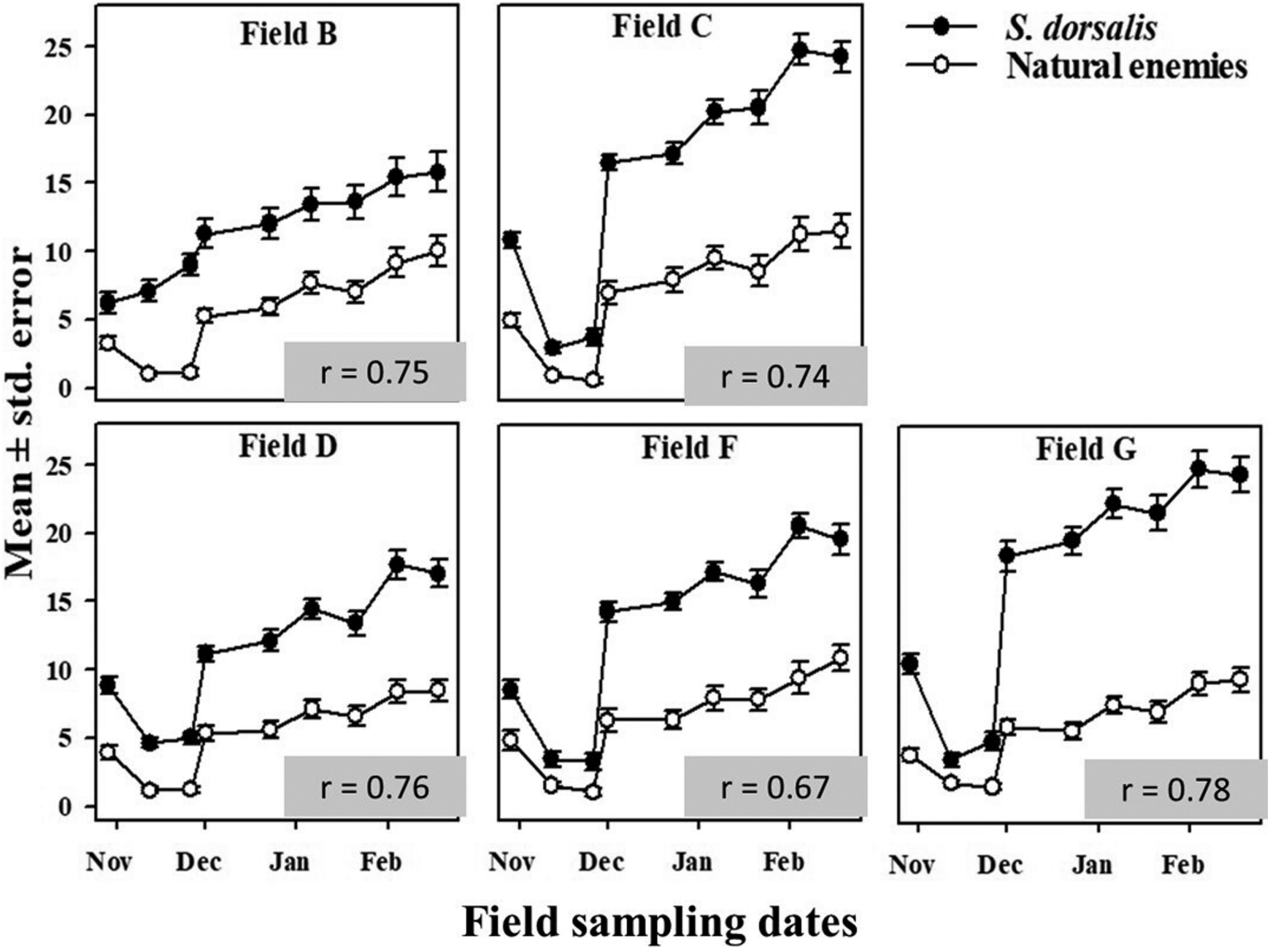
Mean number of *S. dorsalis* and natural enemies sampled at 5 commercial strawberry fields in Florida during 2020–2021. Inset *r* values describe correlation between average weekly captures of natural enemies and mean weekly counts of *S. dorsalis*.

Among natural enemies, dolichopodids were most abundant in sticky traps, followed by *Orius* spp. and *Geocoris* spp. in lower numbers. There was a strong correlation between average weekly captures of natural enemies and mean weekly counts of *S. dorsalis* during both field seasons 2019–2020 and 2020–2021 (Figs. 5 and 6). As observed with *S. dorsalis*, there was a drop in mean weekly captures of predators during November of both years (Figs. 5 and 6). Likewise, a sharp increase in predator captures occurred during December of both years which coincided with the sharp increase of *S. dorsalis* populations.

### Identification of *S. dorsalis* “Hot Spots”

**Fig. 7.**
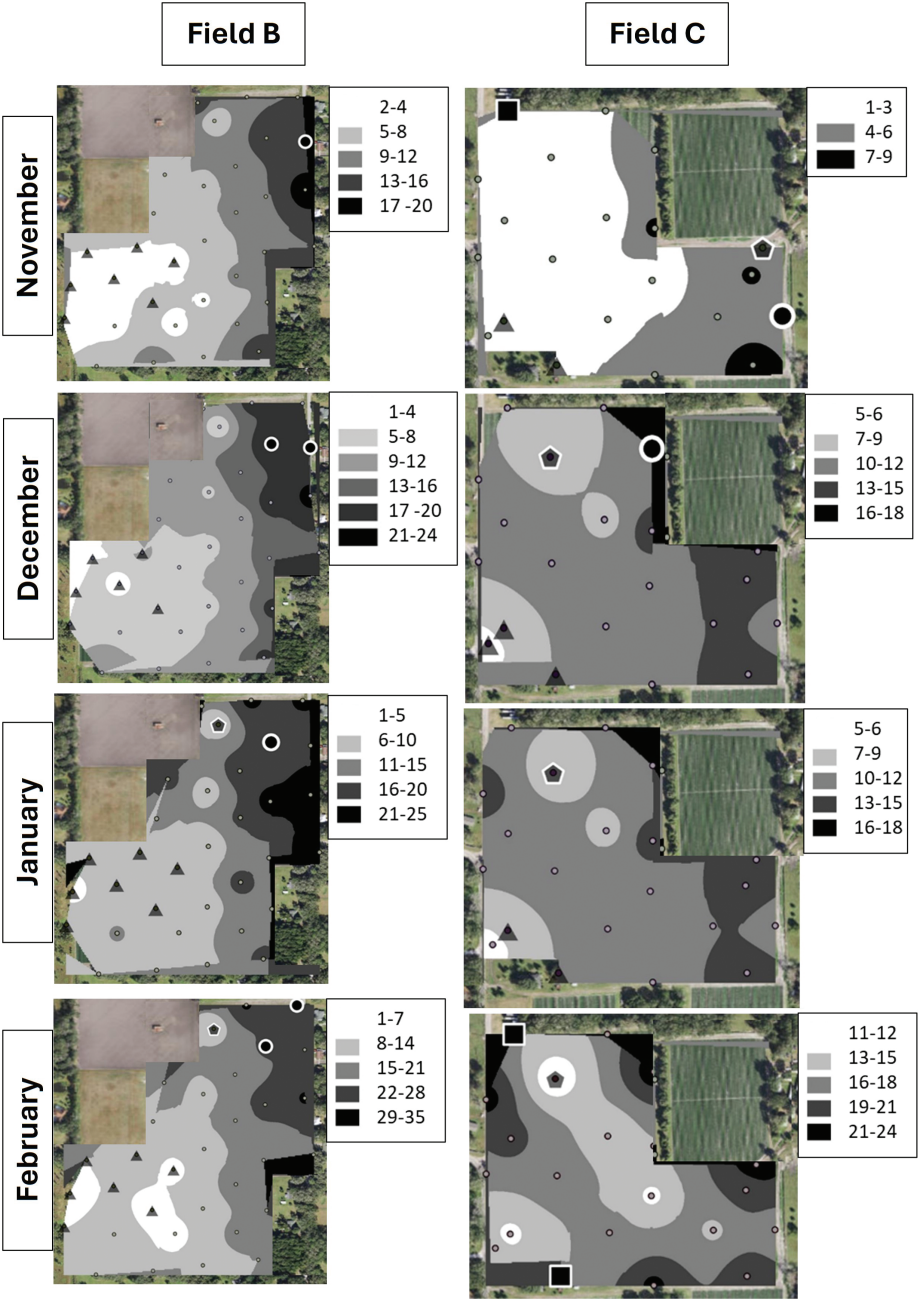
IDW maps plotted in ArcGIS for 2 example strawberry field replicates labeled “B” and “C” sampled over a 4-month period during 2019–2020.

**Fig. 8.**
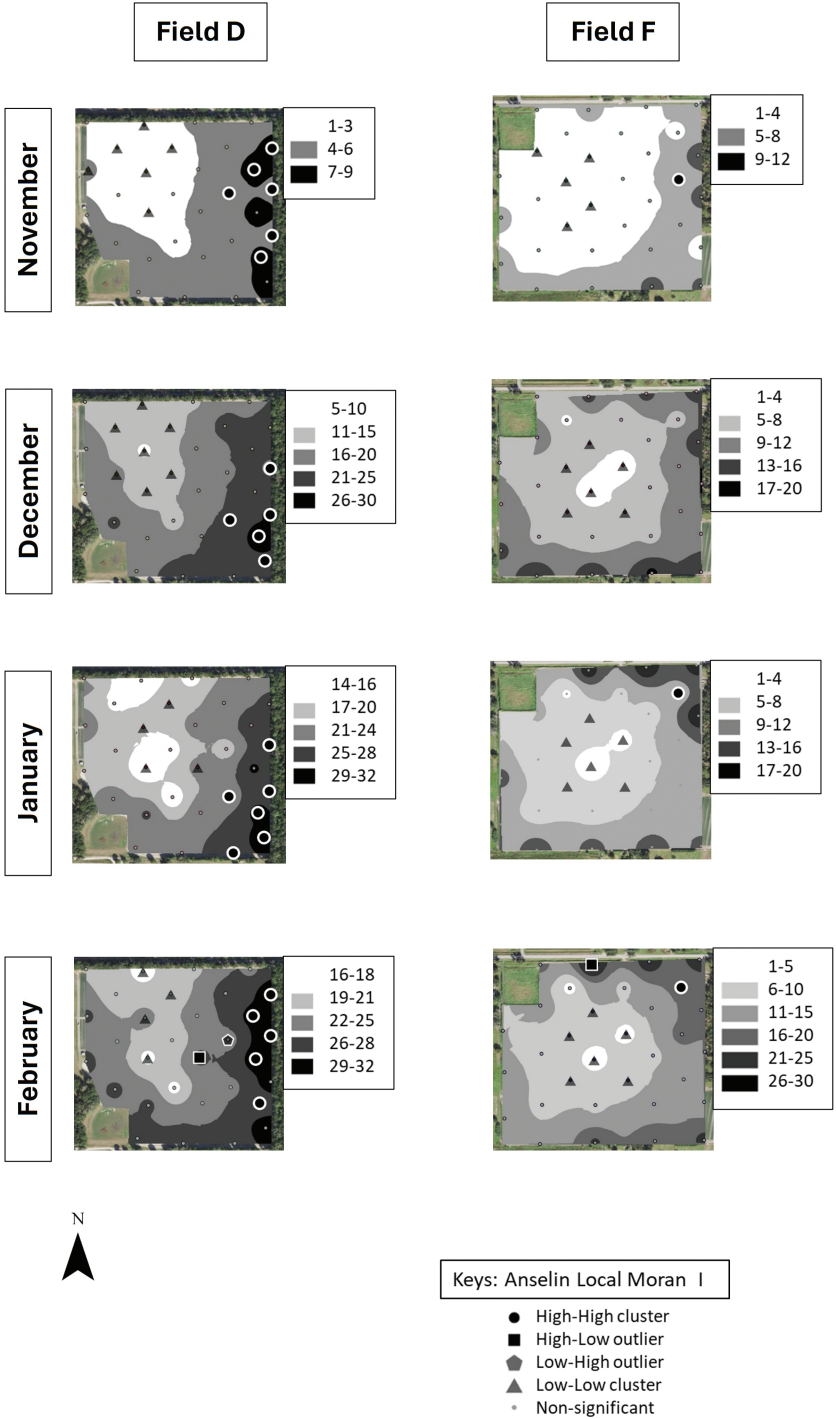
IDW maps plotted in ArcGIS for 2 example strawberry field replicates labeled “D” and “F” sampled over a 4-month period during 2019–2020.

**Fig. 9.**
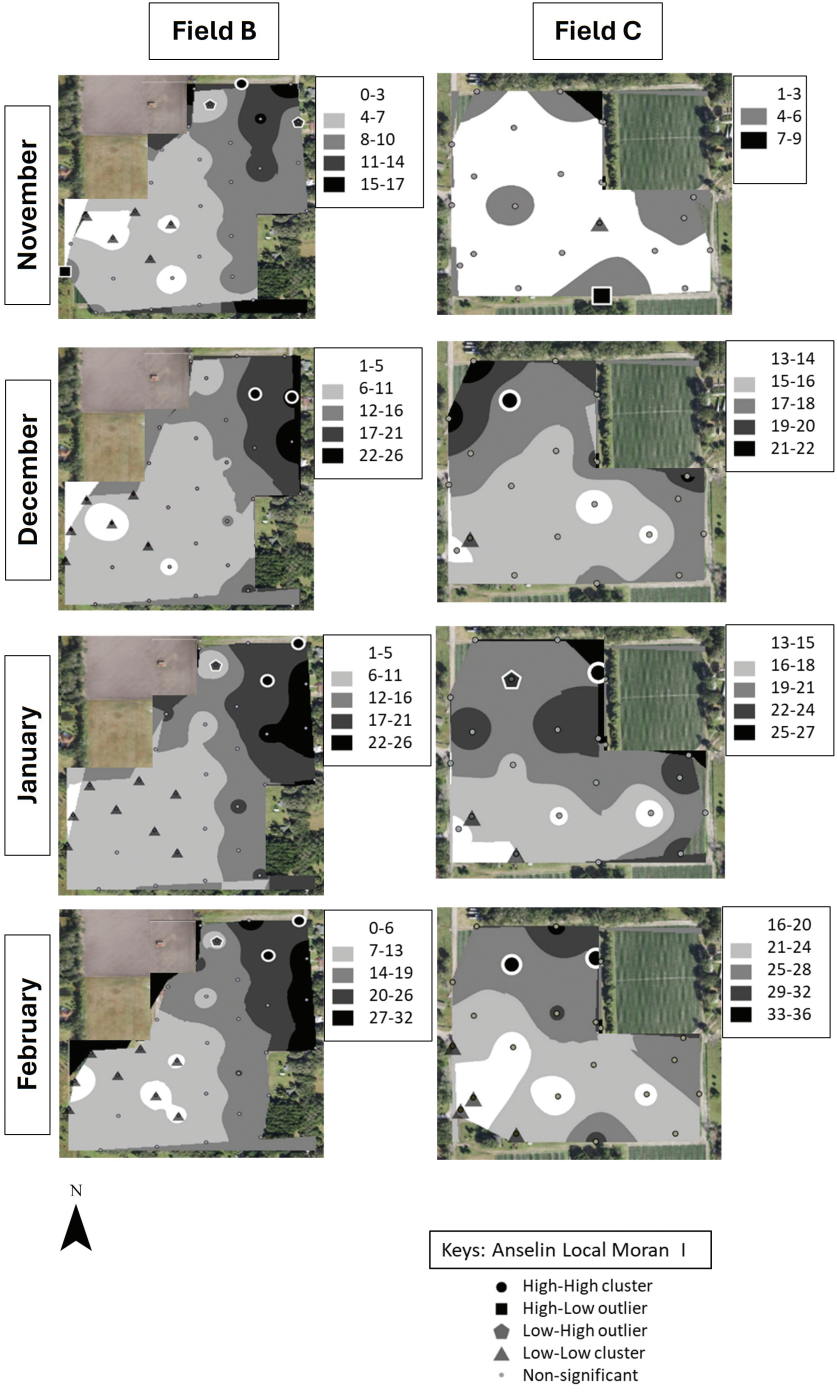
IDW maps plotted in ArcGIS for 2 example strawberry field replicates labeled “B” and “C” sampled over a 4-month period during 2020–2021.

**Fig. 10.**
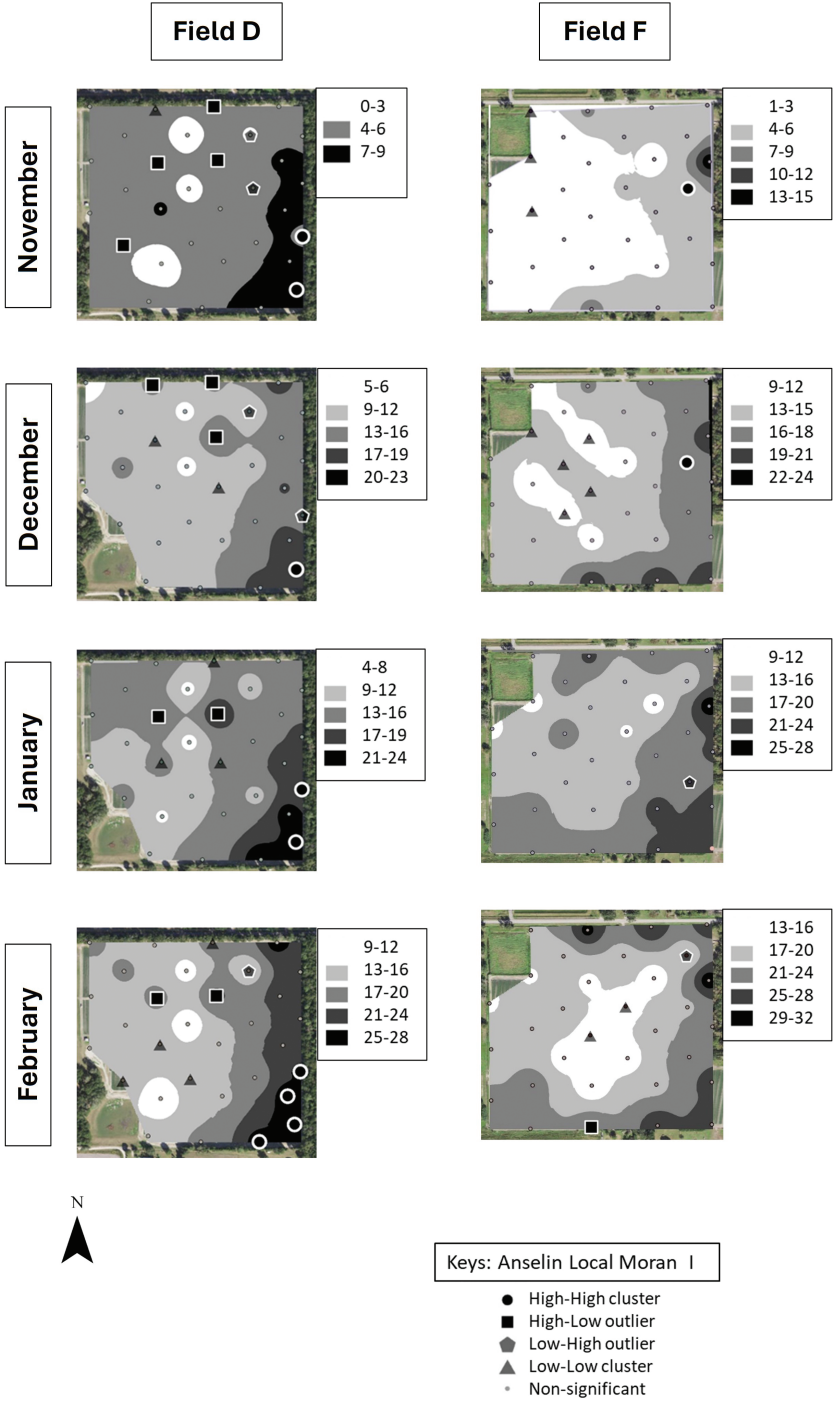
IDW maps plotted in ArcGIS for 2 example strawberry field replicates labelled “D” and “F” sampled over a 4-month period during 2020–2021.

Cluster and outlier analysis showed that *S. dorsalis* formed statistically significant clusters in all 4 sampled fields in 2019–2020 and in 5 out of 6 fields in 2020–2021 (Figs. 7–10). The results showed that high–high and low–low clusters began to appear as early as November in all the fields except 1 in 2020–2021.

**Fig. 11.**
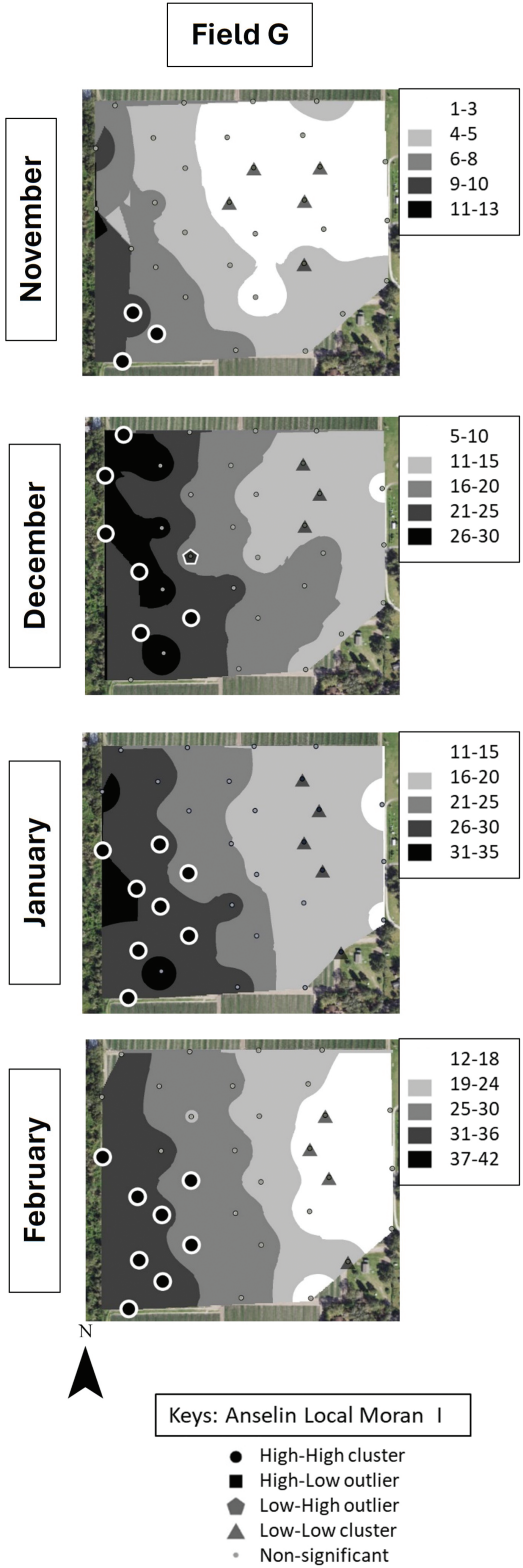
IDW maps plotted in ArcGIS for 1 example strawberry field replicate labelled “G” sampled over a 4-month perio during 2020–2021.

In November, high–high clusters or “hot spots” were formed near field borders during both sampling years except in 2 fields (Figs. 7–10). Although both fields also exhibited hot spots on their borders, there was more inward incursion of *S. dorsalis* in these 2 fields; Field G had several internal high–high clusters including at 100-m inward from the field border. In general, low–low clusters occurred near the center of sampled fields, approximately 300-m inward from the border. Interestingly, “hot spots” that formed in November tended to remain in the same areas throughout the season. For example, Field D contained several significant high–high clusters of *S. dorsalis* from October 2019 that remained consistent through February of 2021 (Figs. 7–10). In general, such consistency among hot spots occurred in other fields as well near borders adjacent to natural areas; however, *S. dorsalis* populations tended to ingress into fields from the field borders as the season progressed but did not reach their highest population densities in the very interior centers of fields (Figs. 7–10). For example, during the 2020–2021 season, several significant high–high clusters of *S. dorsalis* began to occur up to 200-m inward from field borders in Field G by the end of February (Fig. 11).

## Discussion

This is the first investigation to characterize the spatial distribution of *S. dorsalis* in multiple commercial strawberry fields. Our results indicated that *S. dorsalis* form clusters or “hot spot” areas in strawberry fields that remain spatially consistent for up to a 2-year period. Such hot spots occurred mainly along the borders of strawberry fields that were adjacent to natural areas containing potential alternative hosts for thrips and were consistent in their appearance at similar locations within the fields during both years of the study. While we observed the ingress of *S. dorsalis* into field interiors over the course of each season, in general, populations remained much higher on plot borders than interiors.

It is possible that *S. dorsalis* colonize commercial strawberry fields from surrounding noncrop habitats and settle initially on plot borders forming hot spots in the early season. Specifically, *S. dorsalis* are capable of surviving on numerous other crop plants such as ornamentals, fruit, and vegetable crops (Kumar et al. 2013). Similar trends have been observed in other insect pests of various crop systems. For example, in onion (*Allium cepa* L.), onion thrips (*Thrips tabaci* Lindeman) tend to be more numerous near field borders in the early season, and targeting field borders with management sprays is recommended at that time (Shelton et al. 1987, Gill et al. 2015). However, as the season progressed, we observed that high population clusters of *S. dorsalis* could ingress into field interiors, though they did not form high-density hot spots in the central regions of our monitored fields. It is possible that weak flight capabilities prevented *S. dorsalis* from moving more than 200-m inward into the field during the cropping season. Alternatively, there may have simply been sufficient resources available for the clustered populations to remain near areas that were initially colonized. Even though strawberry fields experience heavy foot traffic from manual fruit pickers and runner-cutters or farm vehicles daily within strawberry row beds, the observed spatial distribution patterns we recorded do not support the hypothesis that anthropomorphic movement facilitated hitch hiking by *S. dorsalis* across fields. It thus appears that *S. dorsalis* exhibits an aggregated distribution pattern in strawberry as it does in other crops grown as monocultures such as pepper and potted rose plants (Panthi et al. 2021). It also appears that the pest tends to remain on initially infested plants rather than move to adjacent strawberry plants as they become available, which may also contribute to its aggregated behavior (Panthi et al. 2021). Male and female thrips produce aggregation pheromones, which have been identified in other thrips species, such as *F. occidentalis* and *Thrips palmi* Karny (Kirk 2017). However, no such aggregation pheromone has been identified yet for *S. dorsalis*. Future studies that investigate the potential of an aggregation pheromone in *S. dorsalis* could offer insight into how semiochemicals may affect their distribution patterns in the field and create opportunities for the development of better monitoring traps and potential management tools (Panthi et al. 2021).

There was a consistent low point in seasonal populations of *S. dorsalis* occurring in November/early December during both sampling years. This could be related to decreasing temperatures in FL during that time (Zainab et al. 2016, Priyadarshini et al. 2017) or caused by an increase in insecticide applications against populations of susceptible *S. dorsalis* immigrating into newly planted strawberry fields. The frequency of insecticide applications in strawberries is typically highest during this period in Florida because newly planted strawberries are at a particularly susceptible stage for infestation by *S. dorsalis* (Seal and Klassen 2005, Lahiri 2023). Also, early-season *S. dorsalis* populations typically have no or little prior exposure to insecticides and are thus highly susceptible to insecticide application as they move from surrounding areas into strawberry fields (Kaur et al. 2023). However, by December, surviving *S. dorsalis* populations occurred at much higher population densities. It is possible that management sprays are simply insufficient to mitigate population increases. There is also some evidence that population-wide susceptibility of *S. dorsalis* to insecticides may decrease over the course of a single growing season among rapidly developing and overlapping generations, resulting in higher populations later in the season (Kaur et al. 2023). Our results are consistent with previous findings showing that *S. dorsalis* populations continued to increase over the course of each growing season despite intensive insecticide applications applied in these commercial fields (Kaur et al. 2023).

There was a strong correlation between the average weekly counts of *S. dorsalis* and that of several taxa of potential predators during both sampling seasons. The data suggest that fluctuations in populations of *S. dorsalis* may have driven associated populations of the 3 predator groups enumerated in a typical sinusoidal predator–prey relationship. The average weekly counts of natural enemies were lower than that of *S. dorsalis*; however, the differing sampling methods (leaf + flower samples versus captures on sticky traps) may account for this difference. Within the 3 groups of predators counted, the mean weekly captures of *Orius* spp. and *Geocoris* spp. were much lower than that of dolichopodid flies. Similarly, Krey et al. (2020) reported that dolichopodid flies were the most abundant predators in strawberry fields, and gut-content analysis revealed that their diet consisted of *Drosophila* spp.

Also, the distribution pattern of predators followed that of *S. dorsalis*, with the highest counts recorded on traps deployed on field borders surrounded by unmanaged vegetation and progressively lower captures away from plot borders and inwards into strawberry fields. It is well documented that *Orius laevigatus* and *Orius limbatus* significantly suppress *S. dorsalis* and *Scirtothrips inermis* Priesner on strawberry grown in greenhouses (Mouratidis et al. 2023). Therefore, it may be possible to enhance ecosystem services provided by naturally occurring *Orius* spp. in Florida to suppress *S. dorsalis* populations with conservation biological control. The low captures of predator species on traps in these strawberry fields could have been caused in part by intense insecticide applications for *S. dorsalis* management (Biondi et al. 2012, Roubos et al. 2014). This possibility highlights the need for evaluating other methods, such as planting flower strips or companion plants, that may contribute to the conservation of biological control agents as part of thrips management in Florida strawberries instead of sole reliance on chemical control methods (Kumar et al. 2013).

Initial insights into the annual distribution patterns of *S. dorsalis* derived from this investigation could be useful for the development of precision agriculture practices. For example, sampling and management practices could be targeted to areas adjacent to field borders and no more than 100-m inward into strawberry fields. Furthermore, early season monitoring efforts to detect an initial infestation in November could target borders and surrounding nonhost vegetation. In addition, reserving the use of insecticides against *S. dorsalis* around plot borders could leave insecticide-free refuges for natural enemies in field interiors, similar to the beetle banks used for conservation biological control in other cropping systems (Bianchi et al. 2006). This method could both increase populations of beneficial predators and reduce input costs of insecticides against *S. dorsalis*. Similar practices have been developed for the management of brown marmorated stink bug, *Halyomorpha halys* Stål (Hemiptera: Pentatomidae), in US peach orchards where only field edges were sprayed with insecticides instead of the entire orchard. This strategy was successful in managing *H. halys* while simultaneously reducing insecticide use by 25%–61% as compared to conventional programs (Blaauw et al. 2015). Finally, our cluster analysis revealed the existence of population hot spots that could reappear in the same locations over successive generations. It is possible these data indicate specific suitable microclimates for *S. dorsalis* or the existence of adjacent alternate hosts from which *S. dorsalis* immigrate to infest the newly planted strawberry. This opens the possibility of cultural control methods such as the removal of alternative hosts near crop borders or the placement of strawberry plantings such that they are not located directly adjacent to natural areas. Furthermore, the detection and subsequent precision-guided elimination of such hot spots could have particularly significant impacts on the overall levels of *S. dorsalis* in strawberries and allow growers to effectively manage large areas with an emphasis on monitoring for hot spots rather than area-wide application of insecticides.

Collectively, our results suggest that plot borders could be targeted for the management of *S. dorsalis* populations in strawberries, which could reduce inputs and conserve natural enemies. However, future field assessments are needed to verify the efficacy of border treatments versus whole-field insecticide applications. The role of woodland border vegetation as a source of alternative hosts for *S. dorsalis* also needs to be assessed, given that “hot spots” appeared to develop more frequently on borders of strawberry fields adjacent to woodlands than on borders adjacent to main roads or residential areas.
